# Antioxidant Property Enhancement of Sweet Potato Flour under Simulated Gastrointestinal pH

**DOI:** 10.3390/ijms13078987

**Published:** 2012-07-19

**Authors:** Kim Wei Chan, Nicholas M. H. Khong, Shahid Iqbal, Imam Mustapha Umar, Maznah Ismail

**Affiliations:** 1 Laboratory of Molecular Biomedicine, Institute of Bioscience, Universiti Putra Malaysia, 43400 UPM Serdang, Selangor, Malaysia; E-Mails: nmhkhong@gmail.com (N.M.H.K.); mustyimam@hotmail.com (I.M.U.); maznah@medic.upm.edu.my (M.I.); 2 Department of Chemistry, University of Sargodha, Sargodha 40100, Pakistan; E-Mail: ranashahid313@gmail.com

**Keywords:** sweet potato flour, antioxidant activity, phenolic content, flavonoid content, simulated gastrointestinal pH

## Abstract

Sweet potato is known to be rich in healthful antioxidants, but the stability of its antioxidant properties under gastrointestinal pH is very much unknown. Hence, this study aimed to evaluate the changes in antioxidant properties (total contents of phenolics and flavonoids as well as antioxidant activity) of sweet potato flour (SPF) under simulated gastrointestinal pH conditions. It was found that the yield of SPF crude phenolic extract increased from 0.29 to 3.22 g/100 g SPF upon subjection to gastrointestinal pH conditions (*p* < 0.05). Also elevated significantly were the total phenolic content (TPC), total flavonoid content (TFC) and antioxidant activity of SPF (*p* < 0.05). In summary, the antioxidant properties of SPF were enhanced under gastrointestinal pH conditions, suggesting that SPF might possess a considerable amount of bound phenolic and other antioxidative compounds. The antioxidant properties of SPF are largely influenced by pH and thus might be enhanced during the *in vivo* digestive process.

## 1. Introduction

Oxidative stress, which is defined as the imbalance between pro-oxidants and antioxidants produced in biological systems, pose huge implications in the process of aging and development of chronic diseases such as arteriosclerosis, cancers and diabetes. Reactive oxygen and nitrogen species formed in excess in any biological system destroy cellular components such as lipids, proteins and deoxyribonucleic acid (DNA) or cause a decrease in the capability of antioxidant defenses [1]. Aside from the normal bodily processes that result in the formation of reactive species, dietary, lifestyle and environmental factors, e.g., smoking, alcohol consumption and infections could accelerate the generation of harmful free radicals [2]. Therefore, antioxidants are needed to not only reduce the level of oxidative stress common in many chronic diseases, but also to serve as adjuvants to other standard therapies in order to provide a synergistic effect in combating chronic diseases [2,3]. Antioxidants could be augmented by dietary supplementation with natural antioxidants preferably from routine dietary items [4–6]. Except for the synergists, antioxidants could be classified into either primary or secondary antioxidants [7]. Primary antioxidants or chain-breaking antioxidants are those which actively inhibit oxidation reactions while secondary antioxidants or preventive antioxidants refer to those which inhibit or slow down oxidation indirectly through mechanisms of iron chelation, quenching of triplet oxygen, *etc*. In this case, phenolic compounds are considered unique as they can exhibit both the properties of a primary antioxidant [7] as well as a secondary antioxidant [8].

Sweet potato (SP) is cultivated extensively for its nutritious value across many regions of the world. It is commonly consumed by a significant number of people as a source of staple food. Sweet potato (SP) is specifically known to contain significant amounts of vitamins and minerals, and is reported to possess rich antioxidant contents especially in the form of phenolics [9–11]. Although SP may contain large amounts of phenolics, not all is biologically accessible. The challenge actuality lies in understanding what these phenolics have to undergo in the digestive tract by humans and what processes are involved until absorbed. Structural modifications as well as alterations in functional properties caused mainly by pH changes along different parts of the gastrointestinal tract could interfere with phenolic actions and, thus, affect bioefficacy. Many studies have pointed out significant influences of gut conditions on the bioaccessibility of phytochemicals [12,13]. However, results of studies on the effects of pH changes on phenolic compounds have not been consistent. Specifically, Vallejo *et al.* [14] and Perez-Vincente [15] reported degradation of phenolics upon subjection to gastrointestinal conditions while D’Archivio *et al*. [13] disagreed with such a concept by suggesting that the phenolics bound to food matrices are usually released under gut conditions which increases their availability for absorption and hence action. Huang *et al*. [16] described that antioxidants such as Trolox and α-tocopherol become more stable under gut pH conditions, hence the ability to retain their activity. We hypothesized that the phenolics and flavonoids of SP are not lost when subjected to simulated gastrointestinal conditions and thus, the present study aimed to investigate such phenomena by simulating gastrointestinal pH changes *in vitro*. The present study also sought to evaluate the effects of gastrointestinal pH conditions on the antioxidant activity of sweet potato flour (SPF). No previous report describing the alteration of phenolics, flavonoids and antioxidant activities upon subjection to simulated gastrointestinal pH conditions have been presented. Findings from this study will lead to better understanding of SPF phenolics upon ingestion and how amenable they are for absorption and other beneficial effects on the body.

## 2. Results and Discussion

### 2.1. Extract Yield, Total Phenolic Content (TPC) and Total Flavonoid Content (TFC)

The yield of crude phenolic extracts of treated (SPF subjected to gastrointestinal pH conditions) and non-treated (control) sweet potato flour (SPF) is presented in Table 1. It is evident that treatment of SPF under simulated gastrointestinal pH elevated the yield of crude phenolic extract significantly (*p* < 0.05). The yield of crude phenolics extract was approximately 11 fold higher upon subjection of SPF to simulated gastrointestinal conditions. As the protective effects of cereal fibers depend on their solubility, the increased extract yield of treated SPF is indicative of improved digestibility of SPF upon ingestion.

Table 2 shows the total phenolic (TPC) and total flavonoid (TFC) contents of treated and non-treated SPF. Generally, TPC and TFC of SPF were all found to be significantly increased (*p* < 0.05) under gastrointestinal pH conditions. TPC of SPF under gastrointestinal pH conditions was found to be elevated two-fold, whereas noticeably, TFC was raised 10 fold. The significant increase in TPC of SPF following simulated gastrointestinal pH treatment is comparable to the findings of Liyana-Pathirana and Shahidi [17] who also reported an average two-fold increase for the TPC of commercial soft and hard wheat flour upon simulated digestion. D’Archivio *et al*. [13] and Ortega *et al*. [18] highlighted that food matrices, interaction of other biomolecules and gut related factors could potentially influence food bioavailability directly or by decreasing polyphenol content in food. A reasonable assumption especially in the context of the current study is that pH changes, specifically acidification, lead to liberation of phenolics bound to food matrices, potentially enhancing bioavailability by increasing the amount of phenolics available for absorption. This is further supported by Liyana-Pathirana and Shahidi [17] and Baublis *et al*. [19] who contend that subjection of studied plant samples to gastric pH treatment increases phenolic compositions and contents. Acidification, as accounted by Kroon *et al*. [20], is able to free esterified phenolic compounds from fibrous carbohydrates. Furthermore, indicative of the nutritional value of SPF, extraction of phenolics and flavonoids under subjection to pH adjustment proved to be a good strategy to improve extractability of polyphenols in SPF. SPF treated with gastrointestinal pH conditions showed a marked increase in extract yield, TPC and TFC, indicating marked stability of SPF-derived bioactive compounds in the gastrointestinal tract.

### 2.2. Antioxidant Activity

**Figure 1:**
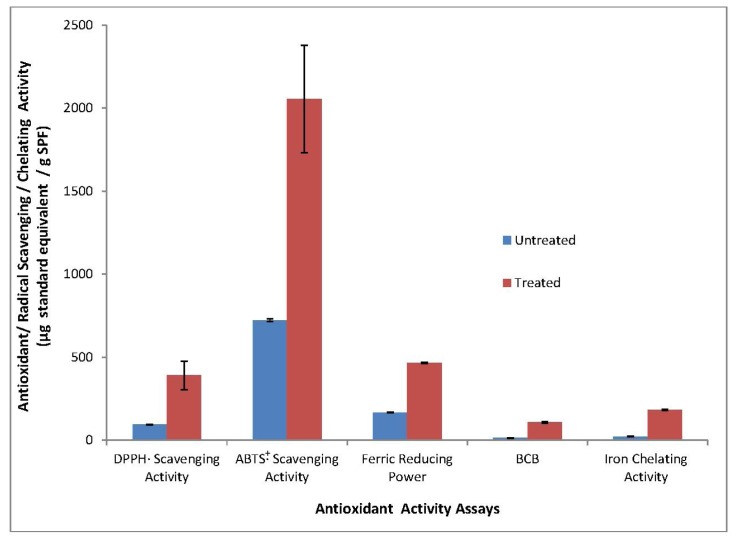
Antioxidant activity of SPF as affected by simulated gastrointestinal pH conditions. Results obtained from means of three determinations ± standard deviation. Trolox was used as a standard in DPPH· scavenging activity, ABTS·^+^ scavenging activity, FRAP and beta-carotene bleaching (BCB) assays. The standard for iron chelating activity assays was ethylenediaminetetra acetic acid (EDTA).

Antioxidant activity of SPF, as affected by simulated gastrointestinal pH conditions, is presented in Figure 1. All five antioxidant assays conducted, concurred that the antioxidant activity of treated SPF was significantly higher (*p* < 0.05) than non-treated SPF. It was found that treated SPF exhibited relatively high 1,1-diphenyl-2-picrylhydrazyl radical (DPPH·) scavenging activity, which is approximately four times the activity shown by non-treated SPF. Correspondingly, 2,2′-azino-bis(3-ethylbenzthiazoline-6-sulphonic acid) radical cation (ABTS·^+^) scavenging activity and ferric reducing antioxidant power (FRAP) assays also showed that treated SPF displayed higher activities (approximately three times higher) than non-treated sample. Treated SPF was also capable of retarding linoleic acid peroxidation eight times more efficiently than non-treated SPF, as indicated through the use of β-carotene-linoleate model system. In the iron chelating assay, treated SPF exhibited chelating capacity eight times higher than non-treated SPF.

Results from all antioxidant assays indicated that upon digestion of SPF, primary antioxidants, particularly phenolic compounds, might be released from the matrices of SPF and depicted by better radicals scavenging activity and ferric reducing capacity in the treated SPF. Primary antioxidants act to scavenge free radicals and interrupt autoxidation pathways by donating hydrogen atoms or electrons so that free radicals can be converted to a relatively stable form of product. Possessing higher affinities for peroxy radicals than lipids, primary antioxidants react prevalently with peroxy radicals which would eventually retard linoleic acid oxidation. This explains the similar increase in the antioxidant activity exhibited by treated SPF through BCB assay. Interestingly, SPF subjected to gastrointestinal pH conditions was also found to display stronger secondary antioxidant properties. Under simulated gastrointestinal pH adjustment, SPF exhibited a higher capability as iron chelator in comparison to its non-treated counterpart. These findings are further supported by the higher TPC and TFC of treated SPF, reported in the former section. Phenolic compounds are important phytochemicals that determine the antioxidant activity in a wide range of botanical samples [21]. Similar improvements in antioxidant capacity were also reported by Baublis *et al.* [19] whereby gastrointestinal pH conditions contributes to a marked increase in antioxidant activity of cereals extract as well. Analogously, the antioxidative activity of polyphenols of different sources and wheat flours too, had been reported to be increased after undergoing pH changes in the gut [17,22,23].

Despite divergent views on the fate of phenolics in gastrointestinal tract, results of antioxidant activity *in vivo* give a fairly consistent result. Phenolics and other antioxidants consumed orally still produce significant effects to the well-being of the body despite undergoing changes in the gut [12,24]. Specifically in human, pharmacodynamics of phenolics would mostly remain unaffected due to enhanced activity under gut conditions [22]. It had been suggested that changes in chemical structure of phenolics and other compounds could have been responsible for improved antioxidant activity rather than its quantity [16]. Additionally, phenolics potency responsible antioxidant activity is suggested to mainly depend on the degree of deprotonation; therefore it is the degree of hydroxyl radical moiety deprotonation, and not the amount of phenolics present, that causes the antioxidant activity [25]. Going by this hypothesis, the improved antioxidant activity recorded upon treatment may also be attributed to the degree of deprotonation caused by the liberated phenolics upon being subjected to simulated digestion of SPF. Accordingly, consumption of SPF could be promoted as the most likely source of phenolics and antioxidants in human where these pro-health factors appear to be stable when subjected to gastrointestinal pH changes. Additionally, nutraceutical ingredients with high antioxidant activity have been proven to dampen food deterioration and lipid peroxidation upon incorporation into common dietary products [26]. SPF which exhibited high antioxidant properties especially upon digestion was particularly promising to be utilized as natural food additive to improve food shelf-life stability as well as human nutrition. Nonetheless, it was suggested that acidification could be considered to be incorporated in the extraction of antioxidants from various food and natural products given its capability to produce a dramatic enhancement of antioxidant activity as compared to conventional extraction.

## 3. Experimental Section

### 3.1. Chemicals

The chemicals used in this study were of analytical reagent or HPLC grade that include: methanol, chloroform and Tween 20 (Fisher Scientific, Loughborough, Leicestershire, UK); linoleic acid, gallic acid, β-carotene (Type I synthetic, 95%), sodium bicarbonate, aluminium trichloride (AlCl_3_), potassium dihydrogen phosphate, dipotassium hydrogen phosphate, 6-hydroxy-2,5,7,8-tetramethylchroman-2-carboxylic acid (Trolox), 2,2′-azino-bis(3-ethylbenzthiazoline-6-sulphonic acid) (ABTS), potassium persulphate, ferrous chloride (FeCl_2_), ferric chloride (FeCl_3_), potassium ferricyanide [K_3_Fe(CN)_6_], ethylenediaminetetra acetic acid (EDTA), 1,1-diphenyl-2-picrylhydrazyl (DPPH), sodium hydroxide (NaOH), Folin-Ciocalteu’s phenol reagent and ferrozine (Sigma-Aldrich Co., St. Louis, MO, USA); n-hexane, trichloroacetic acid and hydrochloric acid (HCl) (Merck, Darmstadt, Germany).

### 3.2. Samples Preparation

Sweet potato (*Ipomoea batatas*) flour was purchased from Sin Guo Co. Pte. Ltd. (Woodlands, Singapore). Two hundred grams of sweet potato flour was mixed with 400 mL of n-hexane and the mixture was homogenized (Ultra-turax T25 basic, IKA^®^-WERKE GmbH & Co. KG, Staufen, Germany) for 15 min at 9500 rpm. Subsequently, the mixture was filtered through Whatman No. 2 filter paper and the residues were re-extracted twice following the same procedure. Defatted sweet potato flour were collected and dried in an oven at 50 °C for 3 h in order to remove the residual solvent. Finally, defatted flours were passed through a 30 mesh sieve and kept at −20 °C prior to the crude phenolics extraction procedure. The finale fat content of sweet potato was 0.73 ± 0.01%.

### 3.3. Crude Phenolics Extraction

Crude phenolics of defatted flours were extracted according to the procedure described by Baublis *et al*. [19]. In brief, 10 g of defatted samples were continuously stirred for 30 min with 150 mL of deionised water. The slurries obtained were centrifuged at 4500 rpm for 30 min at ambient temperature. The resulting supernatants were filtered through Whatman No. 1 filter paper and lyophilised (Virtis Benchtop K Freeze Dryer, SP Industries, Warminster, PA, USA) to obtain the crude phenolics extract. Finally, the yield of crude phenolic extracts was measured before keeping at −80 °C for further analyses.

In order to determine the effect of simulated gastrointestinal pH conditions on phenolic content and antioxidant activity of tested samples, 10 g of defatted flours were continuously stirred for 30 min with 150 mL of deionised water. Then, the mixtures were acidified to pH 2 using 6 N HCl and incubated at 37 °C for 30 min. Subsequently after incubation, the pH was raised to 7 using 4 N NaOH and samples were further incubated for 30 min at 37 °C. Finally, the treated samples were centrifuged, filtered, lyophilized and stored under the same conditions at which untreated samples were placed.

### 3.4. Total Phenolic and Flavonoid Contents

Total phenolic content (TPC) of tested samples was determined by Folin-Ciocalteu Reagent assay. In brief, 10 mg of crude phenolic extracts were individually dissolved in 1 mL of distilled water. Then, 0.1 mL of these solutions was serially reacted with 0.5 mL of 10% (v/v) Folin-Ciocalteu Reagent and 0.4 mL of 7.5% (w/v) sodium bicarbonate solution. After incubation at 40 °C for 90 min, 200 μL of reaction mixtures were loaded into a 96-well plate. The absorbance of the reaction mixtures was read at 765 nm using a microplate reader (Opsys MR™ 96-well microplate reader, Dynex Technologies, VA, USA). Gallic acid was used as standard and TPC of tested samples was expressed in microgram gallic acid equivalents (μg GAE)/g SPF.

Total flavonoid content (TFC) of tested samples was determined using a modified method of Meda *et al*. [27]. Half millilitre of crude phenolic extract solutions was respectively reacted with 2% (w/v) AlCl_3_ for 10 min in a 96-well plate. After that, absorbance of the mixture was measured at 405 nm using a microplate reader (Opsys MR™ 96-well microplate reader, Dynex Technologies, VA, USA). The standard used in this assay was rutin and TFC of tested samples was expressed in microgram rutin equivalents (μg RE)/g SPF.

### 3.5. Antioxidant Activity Assays

#### 3.5.1. DPPH Scavenging Activity

The determination of DPPH radical scavenging activity of tested samples was performed as described previously [4]. In brief, 50 μL of crude phenolic extract solutions, with respective concentration, were reacted with 195 μL of DPPH methanolic solution (0.1 mM) in a 96-well plate. Then the mixtures were swirled gently for 1 min and allowed to stand in dark for 1 h. Finally, absorbance of resulting mixtures was measured using a microplate reader (Opsys MR™ 96-well microplate reader, Dynex Technologies, VA, USA) at 540 nm. Trolox was used as standard and DPPH radical scavenging activity was expressed as μg Trolox equivalent/g SPF.

#### 3.5.2. ABTS·^+^ Scavenging Activity

ABTS radical cation (ABTS·^+^) scavenging activity of tested samples was determined according to the procedure described by Re *et al*. [28] with slight modifications. ABTS·^+^ was produced by reacting 50 mL of 7 mM ABTS·^+^ stock solution with 50 mL of 2.45 mM potassium persulfate for 24 h in dark at room temperature. Then, the ABTS·^+^ solution was diluted with ethanol to an absorbance of 0.70 ± 0.02 at 734 nm (Pharmaspec uv-1700, Shimadzu, Kyoto, Japan). Subsequently, 950 μL of the adjusted solution was reacted with 50 μL of crude phenolic extract solutions. The mixtures were vortexed and allowed to react in dark at room temperature for 10 min. Finally, absorbance of the reaction mixtures was read at 734 nm using a spectrophotometer (Pharmaspec uv-1700, Shimadzu, Kyoto, Japan). Trolox was used as standard and ABTS·^+^ scavenging activity was expressed as μg Trolox equivalent/g SPF.

#### 3.5.3. Ferric Reducing Antioxidant Power (FRAP)

Ferric reducing antioxidant power of tested samples was measured as described previously [21]. Briefly, 1 mL of crude phenolic extract solution was respectively mixed with 2.5 mL of 0.2 M potassium phosphate buffer (pH 6.6) and 2.5 mL of 1% (w/v) potassium ferricyanide. The mixtures were incubated at 50 °C for 20 min followed by addition of 10% (w/v) trichloroacetic acid (2.5 mL) and centrifugation at 3000 rpm for 10 min at ambient conditions. Finally, 2.5 mL of supernatants was diluted with equal amount of distilled water and was reacted with 0.5 mL of FeCl_3_ (0.1%). Finally, absorbance of the reaction mixtures was read at 700 nm using a spectrophotometer (Pharmaspec uv-1700, Shimadzu, Kyoto, Japan). Trolox was used as standard and FRAP of tested samples was expressed as μg Trolox equivalent/g SPF.

#### 3.5.4. Beta-Carotene Bleaching Assay (BCB)

Antioxidant activity of tested samples was evaluated by BCB assay, as described earlier [4]. In brief, 3 mL of β-carotene solution (1 mg β-carotene/10 mL chloroform) were added to 120 mg of linoleic acid and 1200 mg of Tween 20. The mixture was mixed thoroughly and dried under a stream of nitrogen. Immediately, 100 mL of distilled water were added to the dried mixture to form a β-carotene-linoleic acid emulsion. In order to determine the antioxidant activity of tested samples, 1.5 mL of emulsion were added to 20 μL of crude phenolic extract solutions followed by incubation, in a water bath, at 50 °C for 1 h. Finally, absorbance of the reaction mixtures was read at 470 nm using a spectrophotometer (Pharmaspec UV-1700, Shimadzu, Kyoto, Japan). Trolox was used as standard and antioxidant activity was expressed as μg Trolox equivalent/g SPF.

#### 3.5.5. Iron Chelating Activity

Iron chelating activity of tested samples was determined following a method described previously Wang *et al*. [29]. Initially, 1 mL of each crude phenolic extract solution was mixed with 50 μL of 2 mM FeCl_2_ followed by addition of ferrozine (100 μL; 5 mM) in each mixture. The mixtures were thoroughly vortexed and allowed to stand at room temperature for 10 min. After incubation, absorbance of the resulting mixtures was measured at 562 nm spectrophotometrically (Pharmaspec UV-1700, Shimadzu, Kyoto, Japan). EDTA was used as standard and iron chelating activity was expressed as μg EDTA equivalent/g SPF.

### 3.6. Statistical Analysis

Data was reported as mean ± standard deviation from triplicate determinations. Analyses of variance (ANOVA) together with LSD and Tukey tests (SPSS for Windows, Version 15; International Business Machines Corp. IBM: New York, NY, USA; 2006) were conducted to identify the significant difference between the samples (*p* < 0.05).

## 4. Conclusion

The extract yield, total phenolic content (TPC), total flavonoid content (TFC) and antioxidative activity was significantly enhanced when sweet potato flour (SPF) was subjected to simulated gastrointestinal pH treatment. The pH adjustment applied to simulate gut digestion could have improved extractability of antioxidants from SPF. Sweet potato is potentially feasible to be developed as functional food ingredients and nutraceutical additives as their active components might be retained and improved in the biological systems.

## Figures and Tables

**Table 1:** Extract yield of sweet potato flour (SPF) as subjected to simulated gastrointestinal pH conditions.

Sweet Potato Flour (SPF)	Extract Yield (g/100g SPF)
Non-treated	0.29 ± 0.05 a
Treated	3.22 ± 0.65 b

a,b : Results obtained from means of three determinations ± standard deviation. Different letters within the same column indicate significant difference (*p* < 0.05).

**Table 2:** Total phenolic and flavonoid contents of SPF as affected by simulated gastrointestinal pH conditions.

Sweet Potato Flour (SPF)	Total Phenolic Content (μg GAE/g SPF)	Total Flavonoid Content (μg RE/g SPF)
Non-treated	32.54 ± 3.17 a	25.64 ± 4.80 a
Treated	76.98 ± 9.82 b	246.71 ± 91.65 b

a,b : Results obtained from means of three determinations ± standard deviation. Different letters within the same column indicate significant difference (*p* < 0.05).
